# Engaging internal and external audiences to develop and promote zoo-based conservation efforts

**DOI:** 10.1371/journal.pone.0298813

**Published:** 2024-04-17

**Authors:** Nichole L. Nageotte, Marley Steele-Inama, Brittany Frederick, Erica Elvove, Shelby E. McDonald

**Affiliations:** 1 Department of Community Research and Evaluation, Denver Zoological Foundation, Denver, Colorado, United States of America; 2 Women’s Wilderness, Boulder, Colorado, United States of America; 3 Conservation Engagement and Impact, Denver Zoological Foundation, Denver, Colorado, United States of America; Agriculture Research Council, SOUTH AFRICA

## Abstract

As place-based conservation organizations, zoos are in a central position to support individuals in making small changes in their lives that will support the protection of wildlife and their habitats. This paper describes the secondary analysis of data collected from multi-phase front-end, exploratory evaluation that informed the development of a conservation action campaign in association with a non-profit, urban zoo. In phase one, internal organization staff were invited to attend workshops during which they brainstormed potential conservation actions that they felt were important for the zoo to promote. They identified and ranked 164 unique actions. In phase two, the ranking was used to narrow down the 164 actions to 20 actions which were used to develop a survey administered to visitors who opted in to receiving online surveys from the zoo. The survey asked participants to state their interest in each of the 20 conservation actions. The Transtheoretical Model of Behavior Change informed the analysis of responses. Through this approach we identified actions that people were already doing, interested in doing, and not interested in doing. The responses from this survey were used to narrow down the list further to 10 actions used in a survey in phase three. This second survey administered to zoo visitors on grounds asked participants which of the 10 actions they would be most interested in doing, and the perceived barriers and benefits of doing them. This process allowed us to use evidence-based decision making to choose which conservation actions would resonate most with the community for our conservation action campaign. We also were able to identify values visitors held that might influence environmentally friendly behaviors. Visitors who responded to this survey tended to respond in ways that aligned with self-transcendent values. The research suggests that the campaign should focus on habitat restoration and remediation and purchasing wildlife friendly coffee and other products.

## Introduction

The planet is currently facing a biodiversity crisis [1, 2]. According to the International Union for Conservation of Nature (IUCN) Red List of Threatened Species, around 28% of all assessed species including animals, plants, fungi, and chromists are threatened with extinction [3]. These species face a myriad of threats due to human-produced (anthropogenic) pressures such as habitat loss, degradation, and overexploitation; pollution; and concomitant climate change [4, 5]. Thus, human actions play a critical role in addressing current and future threats to biodiversity and there is significant need to understand motivations and barriers for adopting conservation behaviors [6].

Zoos and aquariums (henceforth zoos) are prime locations to promote conservation behaviors, both on individual and collective levels. Worldwide, zoos receive millions of visitors every year. Zoos accredited by the Association of Zoos and Aquariums (AZA) reach around 200 million visitors annually [7]. Given that these institutions intersect with a large audience, zoos are well positioned to make a significant impact on the conservation behaviors of their visitors and nature more broadly. Furthermore, the conservation of wildlife and other conservation practices are often noted in the mission statements of zoos and individuals who visit these places are often already supportive of conservation initiatives [8]. The current paper adds to the knowledge base and ongoing discussions in this area by describing one AZA-accredited zoo’s evidence-based approach to establishing a conservation action campaign (CAC) aimed at driving behavior change and promoting sustainable conservation efforts locally and globally.

### Conservation and human behavior change

Conservation efforts typically center on animal and plant species, yet an increasing number of fields and organizations in the conservation sector are recognizing that addressing *people* and their behaviors is equally important to driving sustainable and positive change [6, 9, 10]. Accordingly, there has been growing interest in the application of human behavior change approaches as well as human social science theory in this area [10–13]. Moreover, the value of human social science research in helping zoos fully achieve their mission has been a topic of increasing discussion, particularly as it relates to zoo-based conservation initiatives [14, 15].

Although there are many models of human behavior change that are commonly used to encourage conservation behaviors, the Transtheoretical Model of Behavior Change (TTM) is particularly applicable to advancing zoo-based conservation initiatives [16–18]. TTM was developed to understand and motivate human behavior change for human health and argues that relinquishing old behaviors and adopting new approaches involves multiple and cyclic stages [19]. Specifically, TTM proposes five stages of change through which individuals progress, regress and cycle: pre-contemplation, contemplation, preparation, action and maintenance [17, 19]. Precontemplation through preparation are considered pre-action stages, whereas action and maintenance stages are considered action-oriented stages. The current multi-phase study used the TTM as a framework to guide the strategy and development of a zoo-based CAC and to understand conservation actions that should be prioritized, adopted, and promoted by the organization through this campaign. This project was conducted in three phases and sought to investigate:

1. Conservation actions that zoo staff, volunteers, and other internal audiences feel are important to promote among external zoo audiences;
2. The willingness and readiness of zoo audiences to engage in various conservation actions;
3. Conservation actions audiences would be most interested in learning more about and the barriers to and perceived benefits of doing that action.

This paper describes the front-end evaluation process and findings that were used to inform the development of a conservation action campaign. Secondary analyses were conducted of data collected over a 6-month period, including in-person workshops with staff, and two surveys of zoo visitors.

## Phase 1 campaign workshops

### Methods

The process of developing our CAC commenced by holding workshops to identify conservation actions the organization could promote with zoo audiences and the wider community. During the workshops, a facilitator used a modified version of the nominal group technique (NGT) described by Smith et al. [20]. NGT is a group decision-making process that is commonly used, particularly in health services industries [21, 22]. NGT has also been applied in conservation research [23]. When used in conservation studies, NGT has been used to guide environmental management decisions, understand stakeholders needs and attitudes, identify capacity needs, and define new concepts in a geographic area or community [24]. NGT has also been applied to guide research and decision-making in zoos [20, 25]. For example, Smith [25] applied NGT with staff and volunteers from three zoos in Australia to identify onsite and offsite conservation behaviors that zoo visitors could take to achieve the aims of formal and informal zoo-based wildlife conservation education programs.

The process of NGT starts with individuals in a group setting independently brainstorming their own ideas; next, each person is given the opportunity to share their ideas without further discussion. Each idea is recorded on a board or flipchart for the group to see [24, 26]. Individually brainstorming ideas and then sharing each idea with the group, without worrying about the ideas being rejected or modified, gives all participants a voice and prevents ideas from being blocked [24]. After all ideas are shared, they are discussed for clarification [24]. After discussion, the ideas are voted on, either privately or as a group [24, 26]. The benefit of this technique is that it allows for a wider variety of ideas to be brainstormed than other methods of engaging in group discussions. Because everyone can share all their ideas, it also promotes equity in the discussion process and reduces the likelihood of one or two people dominating the conversation [26].

#### Workshop participants

All zoo staff and volunteers, as well as graduate students taking classes in association with the zoo, were invited to participate in one of eight, two-hour workshops held in June and July of 2022. Invitations were sent via emails, a digital employee news board, and a volunteer digital newsletter in the spring of 2022. Participants signed up with their names and departments. However, data was not linked to participant names or departments. The CAC workshops were scheduled on different days of the week and at different times (morning, afternoon, and evening) to accommodate varying work schedules. Three workshops took place in June of 2022, and five took place in July of 2022. Each workshop was facilitated by one to three Denver Zoo staff members who were leading the project and associated with the organization’s Conservation Engagement and Impact division. The space in which each workshop was held was set up with up to six tables with four chairs at each table. Participants entered the room and self-selected their tables, which put them in groups of up to four people. The size of the workshop group ranged from nine to 19 participants, with a total of 111 participants that represented all divisions and levels of responsibility at the organization, including four members of the executive team (Table 1).

**Table 1:** Number and percent representation from each Denver Zoo division at all the workshops.

Division Name	Total participants^a^	Percent participants
Accounting/IT	4	3.6%
Administration	1	0.9%
Conservation Engagement and Impact (Field Conservation, Education, Evaluation, Community Engagement)	25	22.5%
Development	3	2.7%
External Relations	6	5.4%
Food Service Contractor	1	0.9%
Graduate students	9	8.1%
Guest Experiences (Guest Operations, Planning, Campus Management)	20	18.0%
Human Resources	2	1.8%
Life Science (Animal Care, Horticulture, Veterinary Medicine, Nutrition)	23	20.7%
Volunteer	15	13.5%
Missing division information	2	1.8%
Total	111	100.0%

^a^There were 336 regular full time and part time paid staff members working during the summer of 2022 (not inclusive of volunteers or temporary staff).

#### Workshop format and data collection

Workshops were facilitated using four guiding questions. Each participant was given a worksheet to assist in helping them think through these questions. The group went through each question together and participants were asked not to jump ahead to the next question until the group was ready to move on. The first question asked, *“What is important for you to protect*?*”* Facilitators explained that participants could think about ecosystems, ecosystem processes, species, or even individual animals when answering this question. Participants provided their responses individually on their worksheets. Participants were informed that responses were from a personal perspective, not from the zoo’s perspective, so they did not have to limit themselves to conservation projects the zoo was currently engaged in. Once participants identified what they felt is important to protect, facilitators asked participants to individually fill out the next question of the worksheet which asked, *“What are the main threats that negatively impact what you want to protect*? *List up to 3 threats*.*”* After everyone finished brainstorming independently, they shared ideas with their small groups. Each person in the group read off the three threats that they identified until each group member had a chance to speak. One group member acted as a scribe, writing each idea on a flip chart. The flip chart page was then posted on the wall near the group so that they could reference what was written for the rest of the workshop.

Next, the workshop facilitator(s) asked that participants brainstorm actions that people can take to alleviate or reduce the threats that were identified. Participants were asked to independently fill out their worksheet under the prompt, “*What are five actions our community can take to alleviate or reduce the threat(s)*?” Participants were informed that they could write five actions that would alleviate one threat, come up with a different action for five different threats, or use a combination of these approaches. They were also told that they could use any of the threats identified by their group in the previous stage; they did not have to focus on the three threats they identified individually. Next, each person shared out five actions they identified with their group and a scribe wrote each action on the flipchart as it was said.

Once all actions were recorded on paper, the facilitators brought all workshop participants back together to share criteria for discussing the actions. These criteria were identified from the literature regarding what influences individuals’ ability and willingness to participate in a new action or behavior. Criteria were that actions should be: 1) new or novel to the audience, 2) easy and accessible, 3) able to be practiced at the zoo or with the zoo in the community, and 4) something that zoo audiences believe will benefit wildlife or conservation efforts [20]. Facilitators also suggested that participants think of the relevancy of the action in terms of personal connections and/or how the individual or community will benefit, as well as how relevant it would be for a large portion of the organization’s audience [11, 27]. These criteria were applied to help participants think through which ideas might be prioritized over others.

The last step of the workshops provided each participant the opportunity to vote on the top five actions they thought the organization should prioritize. First, participants independently filled out the last question of their worksheets to list the five actions they thought were most important based on the discussions they had about the criteria. Once everyone recorded their top five actions, they individually ranked them using a 5-point ranking system (i.e., 5 = most important; 1 = least important). After each person in the group finished the ranking process, the groups reviewed all actions written on the chart paper and recorded everyone’s score for each action. A total score for each action was then calculated by adding all the scores together.

Because the purpose of the activities described in this manuscript were originally intended as a multi-phase front-end, exploratory evaluation to inform program design and internal decision-making, the initial procedures through which data were collected did not meet the definition of research; thus, informed consent was not required. Prior to conducting secondary analysis of the de-identified evaluation data, however, a protocol was submitted to the Heartland IRB and this work was determined to be exempt research (Heartland IRB Protocol Number: 03162023–467).

#### Analysis

The flip chart pages with threats, actions, and action scoring were collected from each group at the end of the workshop. Each action was recorded in a Microsoft Excel spreadsheet. In total, participants identified 605 actions. Many of these were stated by multiple people, so duplicates were removed for a total of 164 unique conservation actions. The total scores for each unique conservation action were also added in the spreadsheet. Scores were calculated by adding the ranking number each participant gave each action. These total ranking scores were added for each code and theme for a total score (see Table 2).

**Table 2:** Action codes and themes, the number of stated actions per code, the number of unique actions per code and the sum of the scores per action for each code.

Code	Description	Number of stated actions	Number of unique actions	Total score
**1.0**	***Support conservation organizations*** ^a^	***53***	***14***	***181***
1.1	Philanthropic funding/donate	7	3	21
1.2	Volunteer/community (citizen) science	26	5	104
1.3	Become member of Denver Zoo or other conservation organization	3	1	11
1.4	Educate others	17	5	45
**2.0**	***Internal outcomes***	**39**	**12**	**132**
2.1	Educate self	19	5	38
2.2	Increase connections with nature or attitudes towards nature	20	7	94
**3.0**	***Help protect wildlife and habitats***	***109***	***27***	***389***
3.1	Conserve, protect, restore, and create wildlife habitats	76	13	295
3.2	Participate in conservation research	1	1	0
3.3	Responsible pet ownership	14	5	28
3.4	Be a wildlife friendly consumer	18	8	66
**4.0**	***Help protect the environment in general***	***286***	***66***	***845***
4.1	Resource sustainability—reduce, reuse, recycle, refuse	103	19	312
4.2	Sustainable purchases	47	15	114
4.3	Reduce carbon footprint	72	16	215
4.4	Reduce water consumption	33	6	145
4.5	Political advocacy/activism (vote, support policy, support candidates, etc.)	31	10	59
**5.0**	***Organizational actions***	***118***	***45***	***383***
5.1	Denver Zoo actions	61	15	212
5.2	Community/business/NGO actions	31	17	96
5.3	Government actions (local, state, federal)	26	13	75

^a^The text in bold and italics is the theme and sums up all codes for each theme.

Qualitative and quantitative data generated during the workshop were analyzed by two of the workshop facilitators (authors 1 and 2) who have backgrounds in social science research. Specifically, a combination of emergent and predetermined coding was used to analyze the lists of unique conservation actions from the flip chart pages that were generated [28, 29]. The process of code development and refinement was informed by prior work in this area [20, 30]. The first and second author met at the end of June to collaboratively code the data from the first three workshops, discussing each action until they reached agreement on the code. Coding consisted of categorizing each unique conservation action by the type of action it is. For instance, the action of keeping cats indoors was coded as responsible pet ownership, along with actions such as keeping cats on leashes and researching exotic pets before purchasing.

Data collected in July were coded by each of these two authors individually, based on the coding criteria established in the previous analysis phase. Authors 1 and 2 then met to discuss the codes. Any differences in coding were discussed until 100% agreement was reached. During this time, the prior code template was revisited to ensure the coding between the two months were aligned. Codes were then further grouped into themes of related conservation behaviors based on similarities in the conservation outcomes of the actions and the audience that might perform them. Finally, total scores for each code were calculated by adding the scores for each of the actions that fell into each code (see Table 2). Total scores for each theme were calculated by adding the scores from each code that fell under it.

### Phase 1 results

The 164 unique actions were grouped into 18 codes and 5 themes (Table 2). The first four themes were actions that individuals could perform. The fifth theme, *organizational actions*, was conservation actions that could only be performed by organizations such as the zoo, governments, or businesses.

The four codes with the greatest number of actions stated were also the same as the codes with the greatest scores. These included: *Resource Sustainability*; *Conserve*, *Protect*, *Restore*, *and Create Wildlife Habitats*; *Reduce Carbon Footprint*; and *Denver Zoo Actions*.

Each of the 164 unique actions was ordered based on the total score, as determined by the participant ranking. We identified the top third endorsed of all actions (56 actions), which were actions with a score of 11 or more. The remaining 108 actions had a score of 10 or less. We then removed the actions from theme five, *organizational actions*. This theme did not align with the overarching goal of the study, which was to identify what the organization could encourage individual visitors and community members to do, not what actions the zoo and other large organizations could or should do. This resulted in 45 top actions, with the total scores ranging from 11 to 187. The average score for these top 45 actions was 31.12, with a median of 22 and mode of 12.

## Phase 2 first survey of zoo visitors

### Methods

The top 45 actions identified through phase 1 procedures were sent to managers and directors associated with the organization’s Conservation Engagement and Impact division and the Environmental Department (n = 18). These teams were asked to choose 15 actions that they felt had the greatest impact on human, wildlife, and/or habitat outcomes *and* that Denver Zoo had the capacity to support or encourage among community members. The leaders were asked to reduce the list of actions to 15 because that number was considered to be manageable based on staff capacity. They recorded their responses in a shared Excel spreadsheet that had each of the 45 actions in rows and each person’s initials in the columns. Each participant recorded their top 15 actions in their respective row/column by placing “1” in their cell for that action. In addition, they ranked the top five out of the 15 actions they chose by highlighting these choices in yellow. The number of times an action made someone’s top 15 list was calculated using the sum function in Excel. The top five preferences were manually counted for each action by counting the number of highlighted cells. These scores were combined with the participant scores for each action from phase 1 resulting in final scores. The top 20 actions identified by these final scores were used to create a survey to send to zoo visitors.

The survey included 34 items that measured intentions regarding each conservation action based on TTM responses, perspectives of the conservation effectiveness of each action, and demographic questions.

Most survey questions were under a heading that read, “*What are your current thoughts or actions regarding the following*?” followed by each of the 20 conservation actions (Table 3). Below is an example of one of the survey items:

**Table 3:** Actions chosen for the survey based on the top 45 actions from the nominal group technique workshops and input from managers and directors of the Conservation Engagement and Impact division and Environmental Department.

Donate money to support wildlife conservation
Volunteer with a wildlife conservation organization
Participate in community-based wildlife conservation research
Learn about threats to wildlife
Learn about ways to protect wildlife
Spend time in nature
Observe wildlife quietly from a distance
Dispose of waste properly when recreating
Plant native plants in yards/container gardens
Install and insect nesting box near your home
Purchase sustainably harvested seafood
Purchase shade-grown coffee
Participate in nature restoration/clean-ups
Drive slower on roads where wildlife crossings occur
Compost organic materials at home
Use reusable containers for storing food
Buy locally sourced food
Eat a mostly plant-based diet
Replace lawn with drought-tolerant xeric plants
Install, or ask your landlord to install, a low-flush toilet

Purchase sustainably harvested seafood

- Never thought about it
- Not interested
- Slightly interested
- Thinking about it
- Planning on doing it at the next opportunity
- I already do this
- Does not apply to me

These response options were based on the TTM stages of change: precontemplation aligned with the first two response options (*never thought about it*; *not interested*); contemplation aligned with the second two response options (*slightly interested*; *thinking about it*); preparation aligned with *planning on doing it at the next opportunity*; and action and maintenance were combined into one response option (*I already do this*). These two stages were combined because they both represent performing the action, which was what was important for this research. A final response option of *does not apply to me* was added with the recognition that not all actions were applicable to all people. Response options were adapted from Dierking et al. [31], and Smith et al. [32].

The next set of questions used maximum difference scaling (MaxDiff). This scale is considered a type of Best-Worst Scaling and is a tool used in marketing research. It works by the participant choosing their favorite and least favorite option from a list [33, 34]. For this study, participants viewed four randomly selected actions. Out of the four actions they saw, they chose the one that they thought would have the greatest positive impact on wildlife and the one that they thought had the least impact on wildlife. They repeated this process five more times for a total of six sets. Because the survey format utilized randomization procedures to choose actions, participants may not have seen every action; however, they may have seen some actions multiple times.

The final section of the survey contained demographic items. Demographic items asked about zoo membership, frequency of visitation, age, children in the household, residency type, and home ownership (Table 4).

**Table 4:** Demographic information collected from participants (n = 401).

**Membership**				
Member		Non-member		No response
68.1%		31.9%		0%
**Visitation**				
0 visits in the past year	1–2 times	3–4 times	5+ times	No response
4.0%	34.4%	27.7%	33.9%	0%

**Age**						
18–25	26–35	36–45	46–55	56–65	66+	No response
2.7%	22.2%	40.9%	17.0%	10.0%	6.5%	0.5%

**Children in the household**				
0 children		1 or more children		No response
36.0%		63.3%		0.7%
**Residency Type**				
Urban	Suburban	Rural	Other	No response
23.4%	68.1%	6.2%	1.5%	0.7%
**Home Ownership**				
Yes		No		No response
75.6%		22.4%		2.0%

Surveys were created in Alchemer and links were sent to 3,192 emails of zoo visitors in August of 2022 who previously had agreed to receive surveys and communications from Denver Zoo. Identifying information was not collected from participants. A total of 468 survey responses were collected (14.7%). Of these, 67 were only partially completed and removed, leaving 401 completed responses (response rate = 12.6%). Only the completed surveys were analyzed.

#### Analysis

Data were exported from Alchemer to SPSS v28.0. Missing data were excluded from Pearson’s chi-square analysis but was included as “missing” in frequencies of responses that were calculated for each item. Pearson’s chi-squares were used to examine associations between demographic groups and their TTM stages of change for each conservation action item. The MaxDiff questions were analyzed by Alchemer by taking the number of times an action was chosen as most effective and subtracting it by the number of times it was selected as least effective. This number was then divided by the total number of times it was an option. Each item was given a score of between -1 and 1, with actions with positive scores being viewed as more effective and actions with negative scores being viewed as less effective [35].

Conservation actions were grouped based on TTM stages of change. Response options were recoded in SPSS to reflect the stages of change. Two of the stages of change were represented by one response option each (preparation and action), two other stages were represented by two response options each (precontemplation and contemplation). This was done to reflect different aspects of these stages. Individuals can be in the precontemplation stage if they are either not interested in doing the behavior or were not aware of the behavior. In the contemplation stage, people could be seriously considering making a change, or merely toying with the idea. After recoding these response options in SPSS, responses to each item could be categorized as precontemplation, contemplation, preparation, action, or not applicable. Percentages of each TTM stage for each action were calculated and compared. Because people can be in precontemplation if they either have never thought about the behavior or are not interested in making a change, we also looked at the frequencies of each response option for precontemplation (*never thought about it*; *not interested*).

### Phase 2 results

#### Chi square associations

Few statistically significant associations were identified. Members were more likely than expected to report that they have donated money (χ^2^ = 24.91, p < .001) and purchased sustainably harvested seafood (χ^2^ = 13.68, p = .003). Specifically, 60% of members donated and 58% of members purchased sustainable seafood compared to 37% of non-members who donated and 46% of non-members who have purchased sustainable seafood.

People in older age groups were more likely than expected to have installed a low-flush toilet (χ^2^ = 41.608, p = .001), with 88% of people 66 or older having done this. Comparatively, rates of low-flush installation ranged from 18% (18–25 year olds) to 70% (56–65 year olds) among other groups, with percentages progressively increasing with increasing age groups.

Whether or not a person had a child at home was associated with several conservation actions. People without children were more likely than expected to have donated money (χ^2^ = 13.245, p = .004), volunteered (χ^2^ = 8.983, p = .03), and learned about ways to protect wildlife (χ^2^ = 12.891, p = .005) than people with children in the home. Specifically, 61.5% of people without children donated money compared to 47% of people with children; 10% of people without children volunteered compared to 4% of people with children; and 66% of people without children learned about ways to protect wildlife compared to 50% of people with children.

Both residency type and housing status were associated with conservation actions. People in rural areas were more likely than expected to have volunteered (χ^2^ = 22.11, p = .009) compared to people from urban or suburban areas. Specifically, 24% of people from rural areas have volunteered compared to 4% of people from suburban areas and 6.5% of people from urban areas. Homeowners were more likely than expected to be in the action stage for composting at home (χ^2^ = 11.159, p = .011) and installing low-flush toilets (χ^2^ = 16.091, p = .001), 36% of homeowners compost at home compared to 21% of non-home owners and 64% of homeowners have installed a low-flush toilet compared to 40% of non-home owners.

#### Conservation actions and TTM stages of change

**Fig 1:**
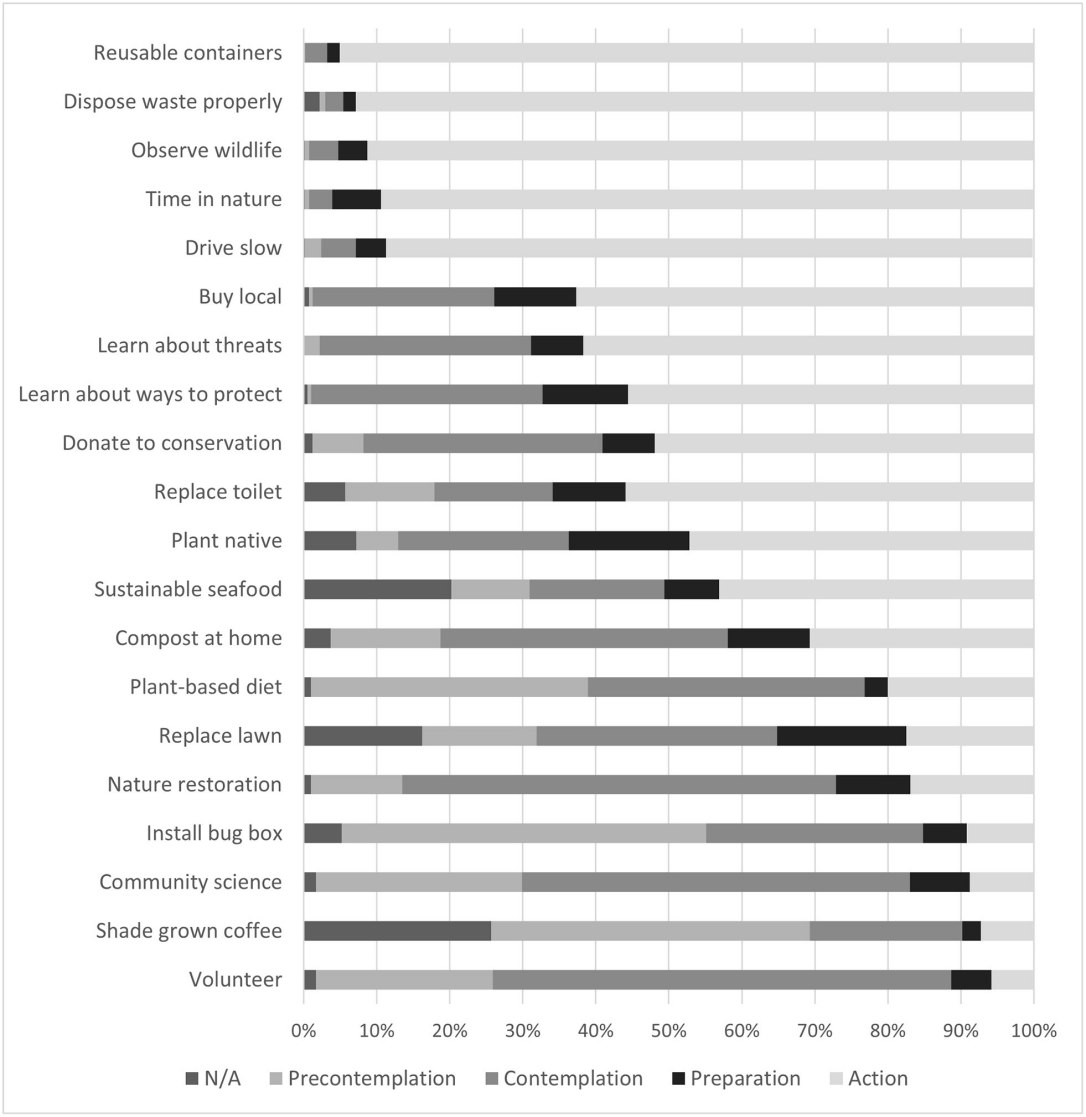
Percent of participants in each TTM stage for each action (arranged by action stage).

The percent of participants in each stage of change for each conservation action can be viewed in Fig 1.

The precontemplation stage represents individuals who are not ready to make a behavioral change. There could be two reasons someone might be in the precontemplation stage for a behavior: either they have never heard of the behavior before and are unaware of the possibility of doing it, or they are not interested in doing the behavior. For the conservation actions in this study that had high percentages of people in precontemplation, we wanted to determine which of these two reasons were most common. This was most noticeable in the actions of buying shade-grown coffee and eating a mostly plant-based diet. In the case of shade-grown coffee, many people were in precontemplation because they had never thought about shade-grown coffee and may have been unaware it was an option (41% never thought about it compared to 3% not interested). For the action of eating plant-based diets, more people were not interested in changing their dietary habits (35%) compared to 3% who had never thought about it.

#### MaxDiff scores

The action that was viewed as having the greatest impact was *Dispose of waste properly when recreating outdoors* (MaxDiff score = .41). Two actions had the same MaxDiff score for the least impact (-0.45). These were *Spend time in nature* and *Purchase shade-grown coffee*. Table 5 presents each action and their respective MaxDiff scores.

**Table 5:** Each action with their rank and MaxDiff scores.

Rank	Action	MaxDiff Score
1	Dispose of waste properly when recreating outdoors	0.41
2	Participate in nature restoration/clean-ups	0.35
3	Replace lawn with drought-tolerant xeric plants	0.30
4	Donate money to support wildlife conservation	0.21
5	Use reusable containers for storing food	0.18
6	Buy locally sourced food	0.16
7	Plant native plants in yards/container gardens	0.14
8	Purchase sustainably harvested seafood	0.12
9	Learn about ways to protect wildlife	0.10
10	Volunteer with a wildlife conservation organization	0.07
11	Participate in community-based wildlife conservation research	0.01
12	Eat a mostly plant-based diet	-0.07
13	Learn about threats to wildlife	-0.09
14	Compost organic materials at home	-0.11
15	Install, or ask your landlord to install, a low-flush toilet	-0.12
16	Drive slower on roads where wildlife crossings occur	-0.22
17	Install an insect nesting box near your home	-0.26
18	Observe wildlife quietly from a distance	-0.28
19	Purchase shade-grown coffee	-0.45
20	Spend time in nature	-0.45

## Phase 3. Survey 2 with zoo visitors

### Methods

After the survey closed and data were analyzed, the results were presented to the Conservation Engagement and Impact division leadership (six people) in September of 2022. During this meeting, the leadership team discussed the results and used them to influence decisions for conservation actions that the campaign should focus on. Decisions were guided by survey results outlined in phases one and two. In addition to the results of the survey, leadership reflected on where the zoo could have the most influence in assisting visitors and community members in progressing through TTM and what actions aligned with initiatives associated with the zoo and AZA.

Through this conversation, three action categories were selected as priorities for the campaign: creating habitats locally, protecting habitats around the world, and encouraging visitors and community members to support Denver Zoo in their conservation efforts. During this meeting, potential actions that would fall within each category were identified based on the survey results. For creating and protecting local habitats, potential actions identified through this process were composting food waste; installing high-efficiency toilets, showerheads, and/or washing machines; planting native gardens; providing shelter for native insects; providing shelter for native birds; and restoring/cleaning habitats in the community. For protecting habitats around the world, the specific actions that were identified were: purchasing wildlife-friendly coffee, tea, and chocolate; purchasing sustainably harvested wood and paper products; purchasing sustainable palm-oil; purchasing sustainable seafood; and donating to support Denver Zoo’s field conservation projects around the world.

In order to ensure that the priorities established through this process were aligned with zoo visitor interests and values, a second survey was created to identify which actions people were most interested in learning about, as well as perceived benefits and barriers to doing those actions. The survey was conducted using visitor intercepts on Denver Zoo grounds. Continual ask sampling was used by drawing an imaginary line on the ground and approaching the next perceived adult (18+) to cross the line using methods adapted from Moss et al. [36] and Clayton et al. [37]. Trained data collectors approached visitors; if that person agreed to take the survey, the data collector waited until the person finished the survey before approaching the next person. Data were collected over a two-week period in November of 2022.

The survey included a list of 10 conservation actions. Participants were asked to select one that they would be most interested in learning more about. After choosing one action, participants were asked to choose a statement that best reflected why they chose that action. The statement options were designed to align with the TTM stages of change: precontemplation, contemplation, preparation, and action/maintenance. Participants were given a list of nine benefits of doing the action they chose (example: it might save money, it is healthy for the environment). These benefits aligned with the four value categories identified by Schwartz [38, 39]: openness to change, self-transcendence, conservation, and self-enhancement. Surveys also asked the participants to choose from a list of seven potential barriers that might prevent them from enacting the action that they chose (for example: not enough time, too expensive). Membership was the only demographic item measured in this survey. No identifying information about the participants was gathered. Surveys were analyzed using frequencies of responses in SPSS.

### Phase 3 results

One hundred and twenty-seven participants completed the survey. Of these, 52% were members of Denver Zoo.

#### Conservation action frequencies

Table 6 shows the percentage of participants (n = 127) who selected each action as the one they would be most interested in learning more about. The most frequently endorsed action was *planting native gardens in your yard/patio*.

**Table 6:** The percent of participants who chose each action when asked which they would be most interested in learning more about (n = 127).

Action	Percent
Planting native gardens in your yard/patios	27.6%
Providing shelter for native birds in your yard/patios	18.9%
Purchasing wildlife friendly coffee, tea, and/or chocolate	14.2%
Restoring/cleaning habitat in your community	11.0%
Composting food waste	10.2%
Purchasing sustainable palm-oil (used in many processed foods)	7.9%
Providing shelter for native insects in your yard/patios	4.7%
How to donate to Denver Zoo wildlife conservation programs	3.1%
Purchasing sustainable seafood	1.6%
Installing high-efficiency toilet, showerhead, and/or washing machine	0.8%

#### TTM stage of change for each conservation action

Around two thirds of participants were in either precontemplation (31%) or contemplation stage (35%) for the actions that they chose. Nineteen percent of participants were in the preparation stage and 15% were in action/maintenance stage.

#### Benefits and barriers

The most frequently endorsed benefits were *it’s healthy for wildlife and/or the environment* (chosen by 74.8% of participants), *it’s the right thing to do* (chosen by 42.5% of participants), and *I might enjoy it or find it rewarding* (chosen by 32.3% of participants). Because participants could choose two actions, the percentages do not equal 100%.

The most frequently endorsed barriers were *financial cost to do the action* (chosen by 46.0% of participants), *not knowing how to do the action* (chosen by 44.4% of participants), *not knowing where to access resources/products* (35.7%), and *limited time to do the action* (chosen by 31.7% of participants).

## Integration and final decision

This paper reports on the methods used to ensure that evidence-based decision making was used in the design of the conservation action campaign. The data from earlier phases of the study informed the later phases. Zoo staff and volunteers brainstormed the conservation actions (phase 1). These actions were narrowed down based on expertise from zoo leadership. Then the inputs of zoo visitors were collected through two surveys (phase 2 and 3). The leaders of the project ultimately used the data from all three phases to make the final decisions regarding the campaign. Two main categories of actions were identified as important for internal and external audiences. These categories resulted in the leadership team deciding to create two campaigns, one focused on creating and investing in local habitats (Campaign A), and one on protecting and investing in habitats around the world (Campaign B). We recommended the campaigns begin by promoting one specific action as specific actions tend to be remembered more than broad actions [40]. Our findings indicated that Campaign A should be centered on developing activities and initiatives focused on restoration and remediation, such as local habitat clean ups, hosting hard to recycle events, and planting native. Results also indicated that Campaign B’s initial call to action should be to encourage consumers to switch to wildlife friendly coffee and chocolate.

## Discussion

This paper presents the evidence-based decision-making process carried out by Denver Zoo to develop a conservation action campaign. Adapting and expanding on social science methodologies first implemented by Smith [25] and Smith et al. [20], our process commenced by engaging zoo staff and volunteers in workshops to identify conservation actions that the zoo’s internal audience felt were important to promote in zoo audiences. This was followed by the development of two surveys that were generated to capture how external zoo audiences feel about specific conservation actions.

The process of gathering feedback from internal and external audiences was critical in leadership choosing actions and behaviors to focus on in upcoming conservation action initiatives. By including both staff and zoo visitors in the process, zoo leadership can be more confident that the initiatives are important and relevant to our key audiences, thus maximizing the potential to support community members in engaging in sustainable behavior change that promotes positive impacts on the environment and wildlife. By engaging internal audiences, leadership can be more confident in the organization’s ability to model the actions they hope to see zoo visitors take in their own lives because they know that there is internal buy-in. Furthermore, these actions might be able to help visitors to connect the animals they see at the zoo with conservation actions that the zoo engages in. Conservation action campaigns tend to be most effective when they can make environmental issues relevant to the audience [27]. For example, the action of planting native gardens was highly endorsed in both surveys. This item might be relevant for the audiences of Denver Zoo due to reduced water availability in the American west. As drought becomes more prevalent [41], it will be more important for people to use native landscaping to reduce water use. These local actions can also be connected to the zoo’s international conservation programs through tailored messaging and programming that emphasizes similarities between local and global conservation issues. With the example of encouraging visitors to plant native plants, this action can be connected to climate change more broadly or invasive species, both of which affect most regions of the world. It is important for organizations to reflect on the conservation issues that are relevant to the audiences they engage with now and in the future.

Obtaining information from zoo audiences was beneficial because it allowed leadership to identify which actions the zoo would be most likely to encourage audiences to progress through the TTM stages of change. For example, one reason why Campaign B will focus on wildlife-friendly coffee and chocolate is that results of the survey suggested there was notable opportunity to build awareness of, and movement toward, encouraging audiences to make sustainable purchases. The results of survey 1 demonstrate that many participants were in the precontemplation stage due to being unaware of this behavior. Because participants reported they were unaware as opposed to not interested, we can assume that there would be limited resistance to this conservation action. Furthermore, there are opportunities for people to learn about how this benefits wildlife. Shade-grown coffee can host greater amounts of biodiversity, especially if the shade comes from native tree types [42, 43]. Many participants in our study did not consider shade-grown coffee to have a positive impact on wildlife. This suggests that many of the participants may not realize the differences in environmental impacts between certified, shade-grown or bird-friendly coffee compared to sun grown coffee. Consequently, there is an opportunity to further connect audiences with this issue by discussing and promoting this action near the relevant animals who live at the zoo. For instance, bird friendly coffee might be promoted within zoo regions with avian species in areas of the world where coffee is grown (e.g. Latin America). Linking zoo animals to local conservation actions in this way might allow for the visitors to better understand the role zoos play in conservation, though this assumption would need to be tested.

### Conservation actions

Three of the conservation action themes identified in phase one were based on the conservation action categories identified by Smith et al. [20]: support conservation organizations, help protect wildlife and habitats, and help protect the environment in general. The first theme, support conservation organizations, captured comments about donations, volunteering for conservation organizations, participating in community science activities, and other similar activities. Our findings diverge from those presented in Smith et al. [20] in that the actions Smith et al. [20] participants identified were zoo-specific. In our case, most of the participants described activities to support general conservation organizations, not just the zoo. On the surface, this may be surprising as participants were all individuals who were associated with the zoo. However, conservation is not a goal that can be achieved by one organization. it takes collective action and Denver Zoo itself has many conservation partners [44]. Many existing conservation action campaigns both in and out of zoos fall under the other themes identified in phase one: 1. protecting wildlife and habitats, and 2. protecting the environment in general [45, 46]. For example, some prior conservation action campaigns in zoos have focused on eliminating balloon releases to protect wildlife that might consume the balloon waste [47], encouraging people to dispose of fishing gear properly to prevent marine entanglement [45], and planting native wildflowers to support pollinators [48].

Another theme of conservation actions that emerged from the data collected during phase one was internal outcomes. Codes within this theme were educate self and increase connections with nature or attitudes towards nature. Specific actions that fell in these codes were things such as spending time in nature and learning about threats to wildlife. Although this theme had the lowest score (see Table 2) among all the themes, this category of actions was still mentioned frequently by participants. This necessitated the need for us to consider how we define a conservation action. Conservation behaviors tend to be actions that people take to either directly or indirectly help wildlife and/or the environment or decrease negative impacts of human-caused threats on wildlife and/or the environment [49]. The theory of planned behavior states that behavior is predicted by intention, which is in turn predicted by attitudes towards the behavior, subjective norms, and perceived control [50]. Since its inception, this theory has been used and refined in many studies to promote conservation or pro-environmental behavior [51]. Increasing positive attitudes towards the environment remain a common outcome of environmental and conservation education [52, 53]. However, there is controversial evidence regarding the strength of association between attitudes and pro-environmental behavior [53–55]. Likewise, knowledge of the environment and environmental behavior is correlated, but do not have a causal relationship [6, 36]. Because of this, we considered these actions of educating oneself and increasing connections and attitudes towards nature, not as direct actions themselves, but as strategies that might lead toward more direct conservation actions down the line. Furthermore, we also recognized that, while we did not see these as conservation actions per se, our participants did, and therefore, it was important to capture that and recognize this theme in our data.

### Values

Findings from our second survey provided information about the values of the zoo visitors. When asked about the benefits associated with engaging in a specific conservation action, most participants stated that the primary benefit was, It’s healthy for wildlife and/or the environment. This aligns with the self-transcendent value category identified by Schwartz [38, 39] which focuses on the welfare of others, including close friends and family, community, humanity in general, and nature. Self-transcendent values are common among zoo visitors [56, 57]. Ballantyne et al. [56] suggested that the self-transcendent values align with zoo missions and actions, which could draw like-minded people to these venues due to shared basic values. This is important to consider when creating educational messaging because certain messages may alienate people of differing values [57]. Knowing that many zoo visitors want to keep animals and the environment healthy will help zoos craft messaging to reflect this. However, zoos also need to be aware that these values may not be shared by every visitor, or the people in our community who do not visit zoos.

### Future research

The results of this multi-phase study will inform the next step of our conservation action campaigns, which is to design programming to promote habitat restoration and remediation and sustainable purchasing of coffee and chocolate. This programming will include developing both activities and messages to promote conservation actions. The message development will include framing, which is a way to make information salient, meaningful, and memorable [58, 59]. To do this, framing connects the message with the audience by providing the information in a way that aligns to the values and ideologies of the audience [60]. Several studies have reported on basic personal values held by zoo visitors [57, 61]. However, zoos are also situated in the cultural contexts of their physical locations. Different regions across the United States have different values systems and ideologies when it comes to wildlife and conservation [62]. Therefore, individual institutions should survey their visitors to understand the values of their personal audiences. It is also important to understand the broader community values and values of people who do not come through the zoo’s doors. Literature that compares values and behaviors of zoo visitors with those who do not visit zoos is relatively scarce (exception includes Packer et al. [61]). Therefore, a necessary area of future research is to explore potential differences between visitors and non-visitors regarding values towards wildlife and willingness to engage in different conservation behaviors. Similarly, different people have different identity-related motivations for visiting, or not visiting, zoos [63, 64]. This study did not explore these identities or how they might influence conservation behaviors. However, other studies have found that identities influence what is learned and behavior intentions [64, 65]. Using social science to investigate community and visitor values and identities could help zoos to encourage conservation behaviors of both these audiences. This, unfortunately, may be a more difficult task for institutions with fewer resources or staff dedicated to social science research and evaluation. University-zoo partnerships may be one avenue through which zoos can collaborate with external researchers to achieve shared goals.

It will also be important to monitor the effectiveness of these behavior change initiatives in zoos, both for visitors and for the zoos themselves. If a goal of zoos is to advance conservation of wildlife and the natural world, they need to be able to demonstrate their successes and identify and learn from what didn’t work. This impact should not only look at how zoos influence audiences, but also the behind-the-scenes conservation work of zoos, such as supporting conservation partners, the field conservation work of the zoo itself, and the zoo’s own sustainability initiatives. This sort of holistic impact measurement of zoo conservation efforts could be highly valuable.

### Limitations

The surveys relied on self-select recruitment methods. For survey one, participants signed up to be part of a mailing list to receive surveys from the zoo. For survey two, participants were intercepted on zoo grounds. These methods do not allow for true random sampling and bias towards individuals who have visited the zoo at least once, and who are willing to take the time to answer the questions. Because of this, we cannot make any generalizable conclusions regarding the conservation actions of our overall community. However, zoo visitors are an important audience for this project and garnering information from them still allows us to make data-informed decisions regarding the conservation action campaigns. We can use this information while recognizing that there are some groups in our community from which we still lack information.

Another limitation is that both surveys used self-reported data. This is a common limitation of measuring conservation behaviors in zoo audiences [36, 40]. Many conservation behaviors are done at home or off zoo property, which makes observations of actual behaviors unlikely or even impossible. In survey one, participants reported on intentions to act and past behaviors regarding each of 20 conservation actions. There is a chance that responses were impacted by social desirability bias if they felt that researchers would be more approving of individuals who have acted, or intend to act, in environmentally friendly ways [66]. However, the impact on social desirability on the data may be inconsequential. When the influence of social desirability bias on self-reporting of conservation behaviors was measured by Milfont [67] they found a weak correlation that was nonsignificant with the removal of a problematic item.

## Conclusion

As zoos aim to be recognized as conservation organizations, it is important to demonstrate the effectiveness of these organizations at making a positive difference for wildlife. For the greatest conservation impact, zoos and other conservation organizations must integrate social science theories and methodologies into their practices. Because most issues facing wildlife and habitats are a result of human action [6], zoos should work with communities and human social structures to change behavior—to benefit both human and wildlife communities that depend upon healthy ecological systems. As Schultz [6] argued, changing human behavior is necessary for the success of conservation, and social science is necessary for changing human behavior.

This paper offers a framework for organizations looking to include the voices of internal and external audiences in decision making and data-informed conservation campaigns. It is logical to assume that various audiences across different regions will have contrasting ideas regarding which conservation behaviors are most relevant and possible to achieve in their community’s unique context. The methods described here can be used and adapted to the needs of different organizations to understand these community perspectives. By including both staff and visitors in the process of deciding what conservation action to focus on, zoos can develop and implement conservation action campaigns that are most relevant and accessible to their internal and external audiences. As zoos continue to design and test programming around conservation actions and related behaviors, it is important that they use data-drive approaches to encourage pro-environmental actions to help all people conserve wildlife.
